# Sugary Cereals at Early Childhood Education Centers Participating in the Child and Adult Care Food Program, 2017

**DOI:** 10.5888/pcd16.180543

**Published:** 2019-03-21

**Authors:** Rebecca M. Schermbeck, Julien Leider, Jamie F. Chriqui

**Affiliations:** 1 Institute for Health Research and Policy, University of Illinois at Chicago, Chicago, Illinois; 2 Division of Health Policy and Administration, School of Public Health, University of Illinois at Chicago, Chicago, Illinois

## Abstract

The objective of this study was to describe the provision of sugary cereals by early childhood education (ECE) centers participating in the Child and Adult Care Food Program (CACFP) before required implementation of the updated CACFP meal pattern standards. We distributed a web-based survey, which included a question on breakfast cereals, to a random sample of 5,483 CACFP-participating ECE centers nationwide. Of the 1,343 centers that responded, 30% did not meet the updated requirement for cereal; 38% of independently owned or operated centers did not meet the requirement. Results indicate the need for additional training and technical assistance on the updated CACFP standards for sugar in cereal.

**Summary**

**What is already known on this topic?**

The meal pattern standards for the Child and Adult Care Food Program (CACFP) were updated in April 2016. One updated standard requires all reimbursable cereals served by CACFP-participating early childhood education (ECE) centers to contain no more than 6 g of sugar per dry ounce.

**What is added by this report?**

Thirty percent of CACFP-participating ECE centers did not meet the sugar-in-cereal standard at the time of this study. Independently owned or operated centers are less likely to meet the sugar-in-cereal standard than centers that are corporate-owned or Head Start–affiliated or that have a food program sponsor.

**What are the implications for public health practice?**

ECE centers that participate in CACFP may benefit from additional training and technical assistance in determining cereals that meet the revised meal patterns.

## Objective

The Child and Adult Care Food Program (CACFP) meal pattern standards were updated in 2016 to align with the 2010 Dietary Guidelines for Americans (1). Effective October 1, 2017, cereals may contain no more than 6 g of sugar per dry ounce (1). Diets low in added sugars are associated with reduced risk of cardiovascular disease, obesity, and dental caries (2). Many children in day care centers consume excessive calories from added sugars (3); approximately 13% of preschool children’s calories are obtained from added sugars (4), exceeding the recommended 10% (2). The limit on sugars in cereal is one method to reduce added sugars for children at CACFP-participating early childhood education (ECE) centers, as ready-to-eat cereal is among the most frequently reported food items on the menus of some centers (5). The objective of our study was to describe the provision of sugary cereals by CACFP-participating ECE centers prior to their year-long transitional implementation of the updated CACFP meal pattern standards, which started on October 1, 2017.

## Methods

Detailed study methods are provided elsewhere (6,7). A web-based survey was developed with input from child nutrition and ECE experts to assess alignment with the updated CACFP meal pattern standards (1), after cognitive testing (6,7) of question wording and format. One survey question asked, “Does your site provide the following cereals [name brand or store brand] to children ages 2–5 years? [Select all that apply]” (6). The response options named 12 cereals (6 of which met the CACFP standard of no more than 6 g sugar per dry ounce and 6 of which did not) and “none of the above.” We classified centers as meeting the updated requirement for cereal if they did not serve any sugary cereals from our list (ie, they served only nonsugary cereals from the list or selected “none of the above”) and as not meeting the requirement otherwise. For each type of cereal, a Likert-type question asked how often the cereal was served (>once per week, once per week, <once per week, I don’t know).

The survey was conducted from August 22 through September 30, 2017. We obtained lists of CACFP-participating ECE centers from licensing agencies in 48 states and Washington, DC; we submitted public information requests in 9 states. From the state-provided lists, we drew a random sample of 5,604 centers nationwide. Of these, 121 centers were determined to be ineligible and were excluded; the final sample included 5,483 eligible centers. Of those, 4,666 (85.1%) were invited via email, 681 (12.4%) were invited via a written letter or combination of letter and email, and the remaining 136 (2.5%) sites either declined by telephone or could not be invited. We obtained completed surveys from 1,343 centers in 47 states and Washington, DC, which resulted in a 24.5% response rate (based on 5,483 eligible centers). The final response rate was comparable to the response rate of other CACFP or ECE center surveys (6–10). The zip code–level socioeconomic and demographic characteristics of survey respondents were similar to those of the entire sample (respondents and nonrespondents) after we weighted to account for nonresponse based on zip code–level characteristics (6). We tabulated unweighted descriptive statistics (numbers and percentages) and weighted percentages and 95% confidence intervals; all percentages in the Results section are weighted.

We conducted logistic regression analyses in Stata/SE version 13.1 (StataCorp LLC), controlling for center-level characteristics obtained from the survey and zip code–level characteristics of the ECE centers. The key outcome measure was whether centers did or did not meet the updated requirement for cereal.

## Results

More than two-thirds of centers (69.9%) reported meeting the updated CACFP requirement for cereal (Table 1). Of the centers that did not meet the requirement, 53.5% served sugary cereals less than once per week, 40.7% served them once per week, and 5.8% served them more than once per week. Most (92.7%) of the centers that did not meet the requirement served a mix of sugary and nonsugary cereals. Roughly one-third (31.3%) of centers reported being independently owned or operated. Nearly two-thirds (64.2%) of centers reported being “very much” familiar with the updated CACFP requirements. Most centers reported compliance checks were conducted by the state (53.9%), were in operation for 10 or more years (61.9%), had 20 or fewer employees (72.3%), and had an enrollment capacity of 51 to 499 children (75.6%). Almost half (49.0%) charged a weekly rate of $101–$200.99 for children aged 2 to 5. Centers were mostly in zip codes where the majority race/ethnicity was non-Hispanic white (56.2%) and that were urban on average (82.7%). The greatest proportion of centers were in the South (42.2%).

**Table 1:** Characteristics of a Sample (n =1,343) of Early Childhood Education Centers Participating in the Child and Adult Care Food Program (CACFP), United States, 2017^a^

Characteristic	Unweighted n (%)	Weighted % (95% Confidence Interval)
**Whether or not center met updated requirement for cereal (≤6 g of sugar per dry ounce)**		
Serve any sugary cereals^b^	379 (28.4)	30.1 (27.4–33.0)
Serve nonsugary cereals only or no cereals listed on questionnaire^c^	955 (71.6)	69.9 (67.0–72.6)
**Independent center (not corporate-owned, Head Start–affiliated, or food program–sponsored)**	431 (32.2)	31.3 (28.6–34.2)
**Have state-enhanced CACFP standards**	671 (50.0)	55.2 (52.3–58.0)
**Compliance checks are conducted by state**	741 (55.3)	53.9 (50.9–57.0)
**Length of time center has participated in CACFP**		
<10 y	440 (37.0)	38.1 (35.0–41.3)
≥10 y	750 (63.0)	61.9 (58.7–65.0)
**Familiarity with revised CACFP standards**		
Not at all/I don’t know	92 (6.9)	7.7 (6.1–9.6)
Somewhat	375 (28.1)	28.1 (25.5–31.0)
Very much	870 (65.1)	64.2 (61.2–67.1)
**No. of employees at center**		
1–10	440 (33.0)	34.3 (31.5–37.3)
11–20	519 (38.9)	38.0 (35.1–41.0)
21–30	211 (15.8)	15.6 (13.5–18.0)
≥31	165 (12.4)	12.0 (10.2–14.1)
**Total enrollment capacity, no. of children**		
1–25	101 (7.7)	7.3 (5.9–9.0)
26–50	235 (17.8)	17.1 (15.0–19.6)
51–100	525 (39.7)	39.8 (36.8–42.8)
101–499	460 (34.8)	35.8 (32.9–38.8)
**Weekly rate for children aged 2–5 y**		
Free/no cost or state subsidized	275 (22.0)	20.4 (18.1–23.0)
$1–$100.99	155 (12.4)	14.1 (12.0–16.6)
$101–$200.99	581 (46.4)	49.0 (46.0–52.1)
≥$201	242 (19.3)	16.4 (14.5–18.6)
**Majority (≥50%) race of population at zip code level**		
Non-Hispanic white	895 (66.6)	56.2 (53.1–59.2)
Non-Hispanic black	111 (8.3)	12.6 (10.4–15.1)
Hispanic	158 (11.8)	14.6 (12.5–17.0)
Mixed	179 (13.3)	16.6 (14.3–19.2)
**Mean % of housing units in urban area at zip code level**	80.5	82.7 (81.1–84.3)
**Census region**		
Northeast	220 (16.4)	17.5 (16.3–18.8)
Midwest	327 (24.4)	18.6 (17.7–19.5)
South	442 (32.9)	42.2 (40.9–43.5)
West	354 (26.4)	21.7 (20.7–22.7)

a Not all categories sum to 100% because of rounding. Denominators for calculation of percentages varied from 1,190 to 1,343. Almost all variation in denominators was caused by missing data due to item nonresponse, except for a small number of observations (1 response for question on weekly rate and 3 responses for question about cereals served) in which the given question was not asked because of a skip pattern. Weighted statistics adjust for nonresponse.

b Sugary cereals named in the survey were Apple Jacks, Frosted Flakes, Froot Loops, Honey Nut Cheerios, Lucky Charms, and Sugar Smacks.

c Nonsugary cereals named in the survey were Cheerios (original), Frosted Shredded Wheat, Kix, Multigrain Cheerios, Rice Krispies, and Shredded Wheat.

ECE centers were significantly more likely to serve cereals that do not meet the updated requirement for cereal if they were independent (vs not independent), “somewhat” (vs “very much”) familiar with the updated CACFP requirements, or charged $1–$100.99 (vs $101–$200.99) weekly (Table 2). Honey Nut Cheerios, served by 23.0% of the ECE centers (sugar content, 9 g per serving [11]), was the most commonly served cereal that did not meet the sugar requirement. In contrast, ECE centers were more likely to serve cereals that did meet the sugar requirements or to not serve any of the cereals listed in our survey if they had 31 or more employees (vs 1–10 employees), were free/no cost or state subsidized or charged $201 or more (vs $101–$200.99) weekly, or were in the West (vs the South).

**Table 2:** Characteristics Associated With Meeting Sugar Requirements for Cereals Served by a Sample (n = 1,343) of Early Childhood Education Centers Participating in the Child and Adult Care Food Program (CACFP), United States, 2017^a^

Characteristic	Any Sugary Cereals		No-Sugar Cereals	
	Adjusted Prevalence, %	Adjusted Odds Ratio (95% CI) [*P* Value]	Adjusted Prevalence, %	Adjusted Odds Ratio (95% CI) [*P* Value]
**Independent center (not corporate owned/head start/food program sponsored)**				
No	30.6	1 [Reference]	69.4	1 [Reference]
Yes	37.7	1.43 (1.02–2.00) [.04]	62.3	0.70 (0.50–0.98) [.04]
**State-enhanced CACFP standards**				
No	34.1	1 [Reference]	65.9	1 [Reference]
Yes	32.4	0.91 (0.67–1.26) [.58]	67.6	1.09 (0.80–1.50) [.58]
**Total enrollment capacity**				
1–25 children	31.2	1.05 (0.43–2.59) [.91]	68.8	0.95 (0.39–2.33) [.91]
26–50 children	35.8	1.33 (0.74–2.39) [.34]	64.2	0.75 (0.42–1.35) [.34]
51–100 children	34.6	1.25 (0.82–1.91) [.29]	65.4	0.80 (0.52–1.21) [.29]
101–499 children	30.3	1 [Reference]	69.7	1 [Reference]
**Familiarity with revised CACFP standards**				
Not at all/I don’t know	37.8	1.45 (0.77–2.74) [.25]	62.2	0.69 (0.37–1.30) [.25]
Somewhat	38.2	1.48 (1.06–2.08) [.02]	61.8	0.68 (0.48–0.95) [.02]
Very much	30.5	1 [Reference]	69.5	1 [Reference]
**No. of employees at center**				
1–10	36.3	1 [Reference]	63.7	1 [Reference]
11–20	35.0	0.94 (0.61–1.44) [.76]	65.0	1.07 (0.70–1.64) [.76]
21–30	27.3	0.63 (0.34–1.15) [.13]	72.7	1.59 (0.87–2.91) [.13]
≥31	22.7	0.48 (0.24–0.98) [.04]	77.3	2.08 (1.02–4.24) [.04]
**Length of time center has participated in CACFP, y**				
<10	36.4	1 [Reference]	63.6	1 [Reference]
≥10	30.6	0.74 (0.54–1.04) [.08]	69.4	1.34 (0.97–1.87) [.08]
**Weekly rate for children aged 2–5 y**				
Free/no cost or state subsidized	19.5	0.40 (0.24–0.67) [<.001]	80.5	2.49 (1.49–4.14) [<.001]
$1–$100.99	46.3	1.57 (1.01–2.44) [.04]	53.7	0.64 (0.41–0.99) [.04]
$101–$200.99	36.3	1 [Reference]	63.7	1 [Reference]
≥$201	23.9	0.53 (0.33–0.86) [.01]	76.1	1.89 (1.17–3.07) [.01]
**Compliance checks are conducted by state**				
No	33.4	1 [Reference]	66.6	1 [Reference]
Yes	32.9	0.97 (0.70–1.35) [.87]	67.1	1.03 (0.74–1.42) [.87]
**Majority (≥50%) race of population at zip code level**				
Non-Hispanic white	32.3	1 [Reference]	67.7	1 [Reference]
Non-Hispanic black	29.7	0.87 (0.52–1.47) [.60]	70.3	1.15 (0.68–1.94) [.60]
Hispanic	37.2	1.28 (0.72–2.27) [.40]	62.8	0.78 (0.44–1.39) [.40]
Mixed	36.3	1.23 (0.76–1.99) [.41]	63.7	0.82 (0.50–1.32) [.41]
**% of Housing units in urban area at zip code level**	—b	1.00 (0.99–1.00) [.60]	—b	1.00 (1.00–1.01) [.60]
**Census region**				
Northeast	36.6	1.03 (0.64–1.64) [.91]	63.4	0.97 (0.61–1.55) [.91]
Midwest	38.4	1.12 (0.75–1.68) [.58]	61.6	0.89 (0.60–1.34) [.58]
South	36.0	1 [Reference]	64.0	1 [Reference]
West	17.7	0.35 (0.22–0.57) [<.001]	82.3	2.85 (1.77–4.59) [<.001]

Abbreviation: CI, confidence interval.

a 1,343 Centers participated in the study; however, results were calculated on the basis of 1,099 centers for which there were no missing data on the outcome variable or covariates. Almost all missing data were due to item nonresponse, except for a small number of observations (3 for the cereals outcome and one for weekly rate) where the given question was not asked due to a skip pattern.

b Because this was a continuous variable it was not possible to compute adjusted prevalence estimates as for the other variables.

## Discussion

To our knowledge, our study is the first nationwide study to report on the provision of sugary cereals to children aged 2 to 5 years at CACFP-participating ECE centers before required implementation of the updated CACFP requirement. Our finding that 30.1% of ECE centers reported not meeting the requirement for sugar in cereal was not entirely surprising because research conducted among ECE centers in North Carolina found that food consumed or served to children aged 2 to 5 years contained excessive amounts of added sugars (3,4). In addition, research suggests that compliance with nutrition requirements and feeding practices may be better among Head Start–affiliated child care sites than CACFP-participating and non-CACFP-participating centers (10,12), possibly because of the nutrition performance standards and nutrition training for staff members associated with Head Start participation (10,12). Our results are similar to those findings in that corporate-owned, Head Start–affiliated, or sponsored ECE centers in our study were more ready than independent ECE centers to implement the new sugar-in-cereal requirement (10,12). This information indicates a need for more training and technical assistance for independently owned or operated centers.

Our study was subject to 4 primary limitations. First, although we used a nationally representative sample, we could not obtain lists of ECE centers from Louisiana or Maine, and no one in Arkansas responded. Second, the study was based on self-reported practices at one point in time. Third, our response rate was only 25%; however, it was similar to the response rate of other surveys of ECE centers (6–10). Finally, the survey did not list every cereal available in the United States, so it is possible that more centers than we reported do not meet the requirement for serving nonsugary cereals.

This study highlights opportunities for training and technical assistance for CACFP-participating ECE centers, particularly independent centers. Future research should assess whether CACFP-participating ECE centers’ compliance with the updated sugar-in-cereal standards improves.
